# Microplastic loads in Eurasian otter (*Lutra lutra*) feces—targeting a standardized protocol and first results from an alpine stream, the River Inn

**DOI:** 10.1007/s10661-024-12791-z

**Published:** 2024-07-06

**Authors:** Ursula Nopp-Mayr, Sarah Layendecker, Marcia Sittenthaler, Matthias Philipp, Ralf Kägi, Irene Weinberger

**Affiliations:** 1 Institute of Wildlife Biology and Game Management, Department of Integrative Biology and Biodiversity Research, University of Natural Resources and Life Sciences Vienna, Gregor Mendel-Straße 33, 1180 Vienna, Austria; 2 Central Research Laboratories, Natural History Museum Vienna, Burgring 7, 1010 Vienna, Austria; 3 Department Process Engineering, Eawag Swiss Federal Institute of Aquatic Science and Technology, Überlandstrasse 133, 8600 Dübendorf, Switzerland; 4 Fondation Pro Lutra, Wasserwerkgasse 2, 3011 Bern, Switzerland; 5 IUCN Species Survival Commission, Otter Specialist Group, Rue Mauverney 28, 1196 Gland, Switzerland

**Keywords:** Particle extraction, Spraint, Sample preparation, Apex predator, Road abrasion, Tire wear

## Abstract

Microplastics (MP) are omnipresent in a wide range of environments, constituting a potential threat for aquatic and terrestrial wildlife. Effects in consumers range from physical injuries to pathological reactions. Due to potential bioaccumulation of MP, predators are of particular concern for MP induced health effects. The Eurasian otter is an apex predator in (semi-)aquatic habitats feeding primarily on fish. Furthermore, the species is classified as “near threatened” on the IUCN Red List. Thus, the Eurasian otter is of conservation concern and may serve as a bioindicator for MP pollution. Feces can be used to detect pollutants, including MP. Initial studies confirmed the presence of MP in otter feces (= spraints). However, as specific, validated protocols targeting at an efficient and standardized extraction of MP from otter spraints are missing, experimental results reported from different groups are challenging to compare. Therefore, we (i) present steps towards a standardized protocol for the extraction of MP from otter feces, (ii) give recommendations for field sample collection of otter spraints, and (iii) provide a user-friendly step-by-step workflow for MP extraction and analysis. Applying this framework to field samples from five study sites along the River Inn (*n* = 50), we detected MP of different sizes and shapes (ranging from microfibers to road abrasion and tire wear) in all otter spraint samples.

**Supplementary Information:**

The online version contains supplementary material available at 10.1007/s10661-024-12791-z.

## Introduction

The presence of microplastics (MP) has been reported worldwide for a broad range of environments, constituting a global environmental problem (Citterich et al., 2023; Jeftic et al., 2009; MacLeod et al., 2021). MP are defined as solid synthetic, not metallic polymer particles that are insoluble in water with sizes from 1 μm to 5 mm (ISO/TR 21960:2020(en)). Due to long-range transport, MP can be found in remote areas like alpine lakes, in polar regions (Zhang et al., 2021) or even as accumulations in oceans (Lebreton et al., 2018). MP have been recorded at different levels of the aquatic food web (Vikas Madhav et al., 2020) including microorganisms (Karlsson et al., 2017; O’Connor, Lally, Koelmans, et al., 2022), fishes (Sequeira et al., 2020), birds (Carrillo et al., 2023; Winkler et al., 2020), and mammals (Philipp et al., 2021).

Several features of MP drive their bioavailability in the ecosystem, including their size, density, abundance, and color (Wright et al., 2013). MP intake by animals might occur through feeding on polluted prey specimen and plants, water filtration, or inhalation of polluted air. Consumption of MP can lead to an accumulation in the gastrointestinal system resulting in starvation, injuries, and/or pathological reactions (Derraik, 2002; Farrell & Nelson, 2013; Welden & Cowie, 2016). Additives, such as UV or heat stabilizers, deliberately added during the synthesis of the plastics or unreacted monomers remaining in the MP can have cytotoxic effects (Hahladakis et al., 2018). Furthermore, due to their (hydrophobic) surface properties, MP can accumulate pollutants like heavy metals, persistent organic pollutants, or antibiotics (Avio et al., 2017; Holmes et al., 2012). These substances can be released from the plastics, especially when exposed to different physical-chemical conditions in the gut of animals (Hahladakis et al., 2018). Apex predators are evaluated as meaningful indicators of MP loading and biomagnification (O’Connor, Lally, Mahon, et al., 2022) and could thus be used for environmental MP pollution monitoring as they frequently roam large home ranges and hunting grounds allowing for MP sampling at large spatial scales (see Carlin et al., 2020; Nessi et al., 2022).

One of the Central European apex predators is the Eurasian otter (*Lutra lutra*), hereafter referred to as “otter,” which inhabits a wide range of (semi-)aquatic habitats (Kruuk, 2006a). Its prey spectrum not only consists of fish, but also includes amphibians, crustaceans, small mammals, reptiles, mollusks, and waterfowl (Krawczyk et al., 2016). During the last century, the otter disappeared from large parts of its native range due to direct persecution, habitat loss, and environmental toxins (Kruuk, 2006b). A ban on several harmful pollutants in the 1990s in addition to the protection by the Bern convention (Bern Convention Appendix II, 1979) and EU Habitats Directive (Council Directive 92/43/EEC of 21 May 1992, Annex II and IV) have contributed to an increase in population numbers in Western Europe and a return of the otter to large parts of its natural habitat (Duplaix & Savage, 2018). Nevertheless, the otter has been recently classified as “near threatened” on the IUCN Red List with decreasing population trend (Loy et al., 2022). Besides degradation of waterbodies, decrease in fish biomass in freshwaters, road kills, and illegal persecution, anthropogenic pollution of aquatic habitats pose a major threat to the otter (Duplaix & Savage, 2018). Even though MP pollution of the otter´s habitat in Central Europe has already been reported, for example from several lakes and rivers (Lechner & Ramler, 2015) as well as in the otter’s prey (Karlsson et al., 2017; Roch et al., 2019), only few studies have addressed and discussed contamination of otters as apex predator of these habitats (e.g., Smiroldo et al., 2019).

Otter feces (so-called spraints) are used to confirm the presence of otters or to study their foraging ecology, but also to detect their exposure to selected pollutants (Nelson et al., 2015). MP were reported in otter spraints for the first time in the course of a dietary analysis in northern Italy by Smiroldo et al. (2019) and Santillán et al. (2020) found MP particles in the feces of the marine otter (*Lontra felina*). Recently, O’Connor, Lally, Mahon, et al. (2022) confirmed the presence of MP in otter spraints in Ireland. However, data on the MP contamination of otters in the alps are still missing and monitoring of MP bioavailability within alpine river systems by means of bioindicators is urgently needed. Unfortunately, these pioneering studies used different methods to extract MP from spraints, hindering comparisons between countries and otter species. A specific, validated protocol targeting at an efficient and standardized extraction of MP from otter spraints is thus required to allow for comparisons of results from different areas or different otter populations and for a standardized monitoring of MP pollution.

In the given study, we present steps towards a standardized protocol targeting at the extraction of MP from otter spraints. Furthermore, we provide recommendations for field sample collection of otter spraints and outline a user-friendly step-by-step workflow for MP extraction and analysis. We apply this protocol to a set of otter spraint samples from five study sites along the course of the River Inn.

## Material and methods

### Study sites

**Fig. 1:**
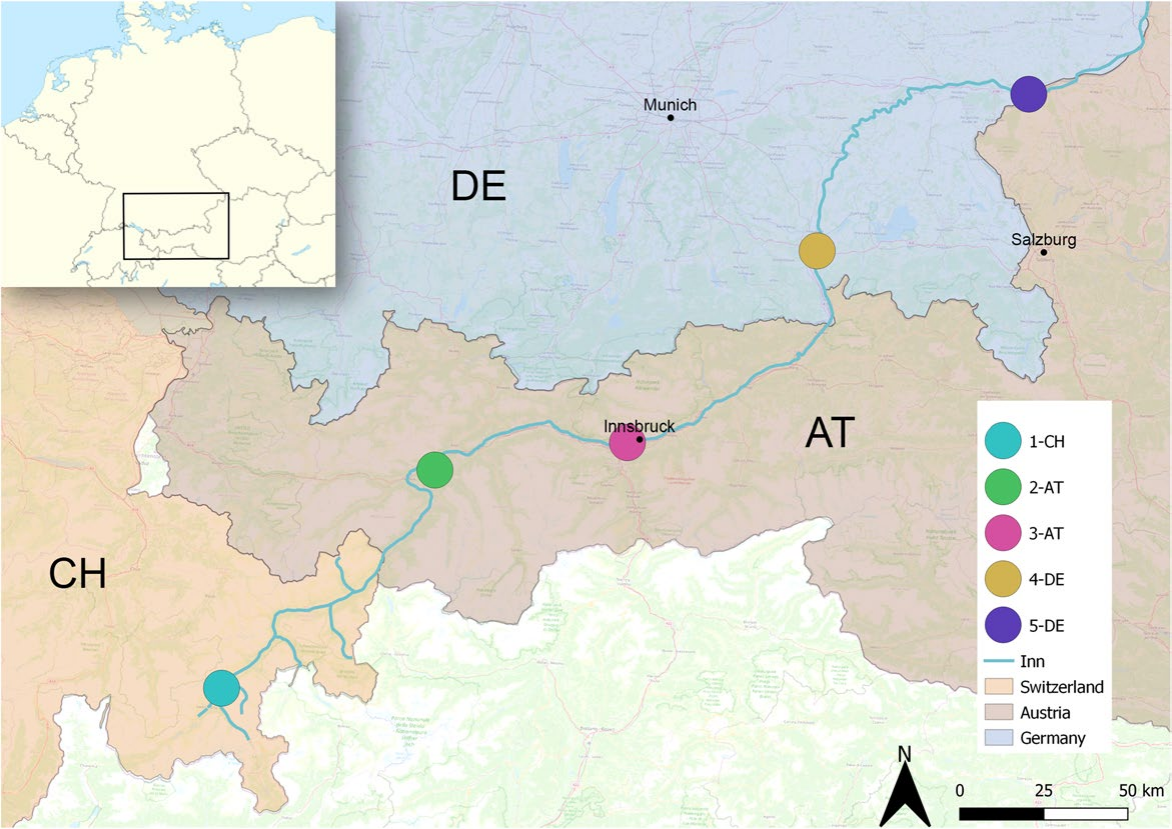
Location of sampling sites along the River Inn in Switzerland (CH), Austria (AT), and Germany (DE)

Otter spraints were collected at five distinct sampling sites along the River Inn, which is one of the longest alpine rivers with a length of 517 km (Fig. 1). It has its source at the Lake Lunghin (46° 25′ N, 9° 40′ E) in the Upper Engadin in Switzerland (CH), passes through Austria (AT) and ends in Germany (DE) in Passau (48°34′ N ,13° 28′ E) where it enters the Danube. The length of the Inn in Switzerland is 104 km, followed by 320 km in Austria and 93 km in Germany.

The riparian stretches of the River Inn show a long history of human intervention with conversions of primal alluvial plant communities to grassland, farm land or settlements (including roads and industrial areas) over long sections of the river. The majority of areas remaining in a near to nature state is located in Switzerland in contrast to Tyrol (AT) with a high proportion of strongly modified surroundings, while the German part shows alternating sections of near to nature and strongly modified stretches. The area “Unterer Inn” (Site Code: AT3119000) is protected under the Ramsar Convention, EU Habitats Directive (92/43/EEC) and considered as a Natura 2000 area. The study sites were situated along the River Inn at distances of approximately 100 km (Fig. 1), representing different river segments (Table S1 in Online Resource 1). The study sites included the riverbanks of the Inn as well as some of its tributaries to account for biologically meaningful hunting ground sizes of the otter (Kruuk, 2006c). The choice of sampling sites was further based on existing documentations of verified otter occurrences (Bayerisches Landesamt für Umwelt, 2021; Kranz & Podelnik, 2013; info fauna CSCF, 2023).

### Field sample collection

We collected ten otter spraints per study site in October and November 2021. As MP are ubiquitous in the environment, it was crucial to prevent contamination of spraint samples by external MP sources (e.g., from the substrate). Thus, spraints from smooth surfaces like stones or concrete were preferred to spraints being positioned on other substrates (e.g., sand and soil). Spraints were collected using aluminum foil (not plastic bags) and stored separately in a clean and dry glass jar. Throughout handling in the field and in the lab, synthetic clothes and work material were avoided. In addition, field blank samples were taken (see also Prata et al., 2019) by placing a piece of aluminum foil next to a random chosen spraint while getting the actual sample. Once the spraint had been collected, the piece of foil was folded and stored the same way as the actual samples. After field collection, samples were stored at – 20 °C until further processing.

### Sample preparation protocol—MP extraction

Targeting at a validated protocol for otter spraints, the procedures for the extraction of MP from organic samples such as seawater or sediment (Masura et al., 2015) and from sewage sludge (Philipp et al., 2022) were compared in terms of their applicability to spraints. The resulting protocol aims at a quantitative evaluation of MP particle loads in otter spraints in terms of numbers, size and shape (= particle types) and the chemical characterization of MP. We differentiated five different particle type classes (see Bertling et al., 2022; Lusher et al., 2020): (1) microfibers, (2) road abrasion particles and tire wear, (3) fragments (formed by degradation of larger materials), (4) pellets (primary MP), and (5) conglomerates (bigger constructs consisting of several items, held together by microfibers). To evaluate the protocol, six test otter spraints (sampled at other regions in Switzerland) were spiked with surrogate standards (see Philipp et al., 2022) to assess recoveries of the extraction protocol.

**Fig. 2:**
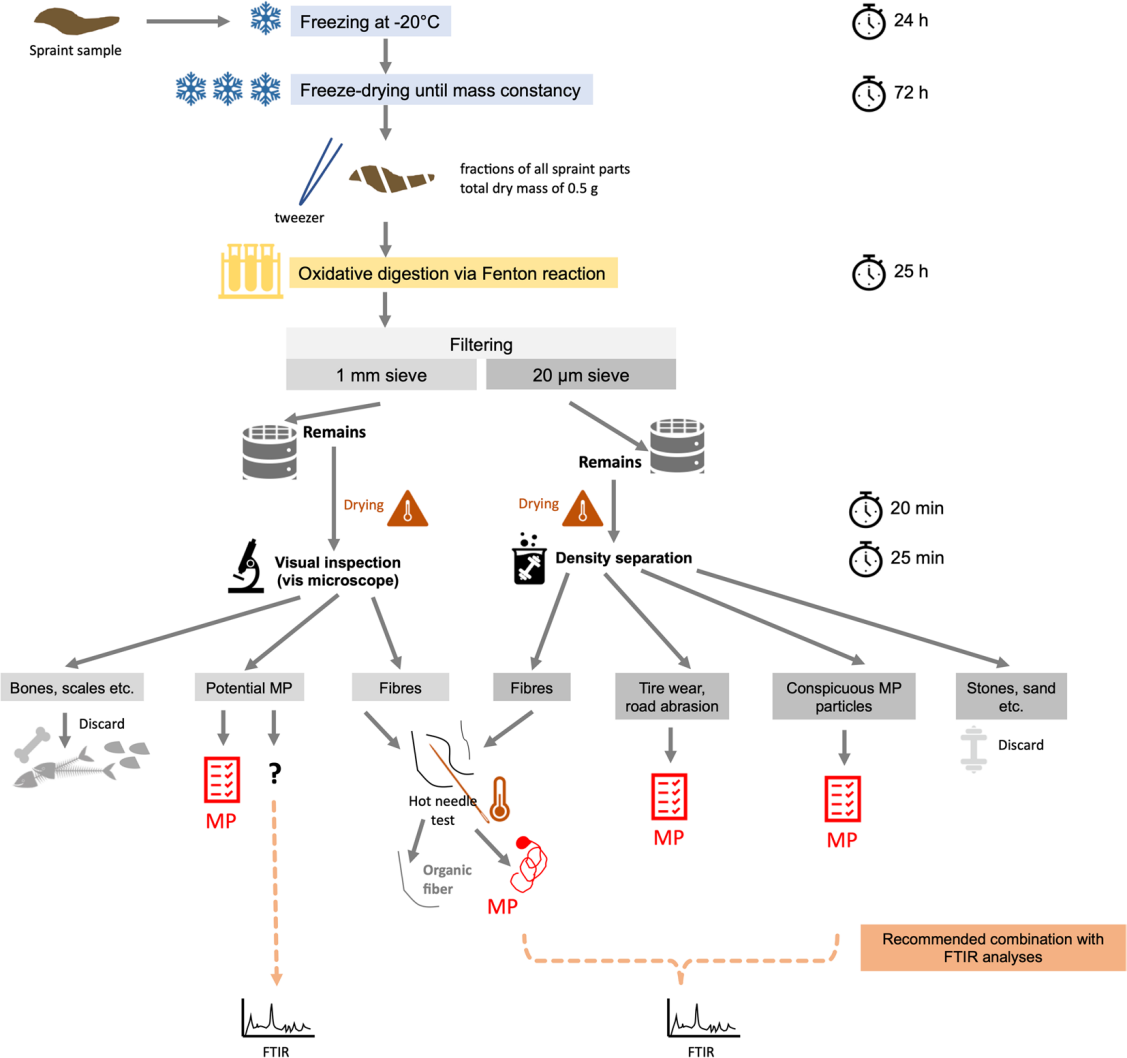
Workflow of otter feces (= spraint) sample preparation for the extraction of microplastics (MP) and quantitative MP determination (expressed in numbers of particles per g dry mass as standard measure)

In the following, we describe the protocol (see also Fig. 2) including the rationale behind the respective procedural steps. For protocol development and the final analyses, we used subsamples with a dry mass of 0.5 g per spraint sample.

Sample preparation included the following steps:

1. *Freeze-drying of spraint samples*

Target: Gaining comparable reference dry weights of samples

- *Step 1:* Preparation of samples for freeze-drying by freezing them for at least 24 h at a temperature of − 20°C in closed glass petri dishes (1 sample per dish)
- *Step 2:* Removing lid of the glass petri dishes, covering them with a paper towel (to avoid air-born cross-contamination), placing them in the freeze-dryer for 72 h (until mass constancy)
- *Step 3:* Storage of freeze-dried samples in closed glass petri dishes in a desiccator until further processing.

*Note:* Accounting for a maximum water content of 41% in our test samples, fresh spraint sample masses should be collected in the field that are 1.7 times higher than the targeted dry mass to ensure sufficient dry sample mass.

2) *Oxidative digestion via Fenton reaction*

Target: Mineralization of non-target, easily digestible organic matter; the protocol refers to a standardized mass of 0.5 g dried spraint sample. Enzymatic digestion as used by Philipp et al. (2022) was not necessary as non-target organic material was sufficiently removed by Fenton reaction in our study.

- *Step 1:* Filling 0.5 g of dried sample mass in 300-ml glass beaker
- *Step 2*: Adding 10 ml DI H_2_0, 20 ml of H_2_O_2,_ 2 ml Fe(II)SO_4_ (7 x H_2_O, 2 mmol/l), 2 ml protocatechuic acid (2 mmol/l) (protocatechuic acid prevents iron (III) particle precipitation)
- *Step 3:* Covering glass beaker with aluminum foil (to avoid evaporation of water and cross-contamination of samples), shaking on a horizontal shaker in a fume hood for 1 h at 100 rpm at room temperature (exothermic reaction); then raising temperature to 40 °C and shaking for 24 h.

3) *Filtering*

Target: Separation of particles into two different size fractions, prevention of masking of smaller particles by larger ones.

**Fig. 3:**
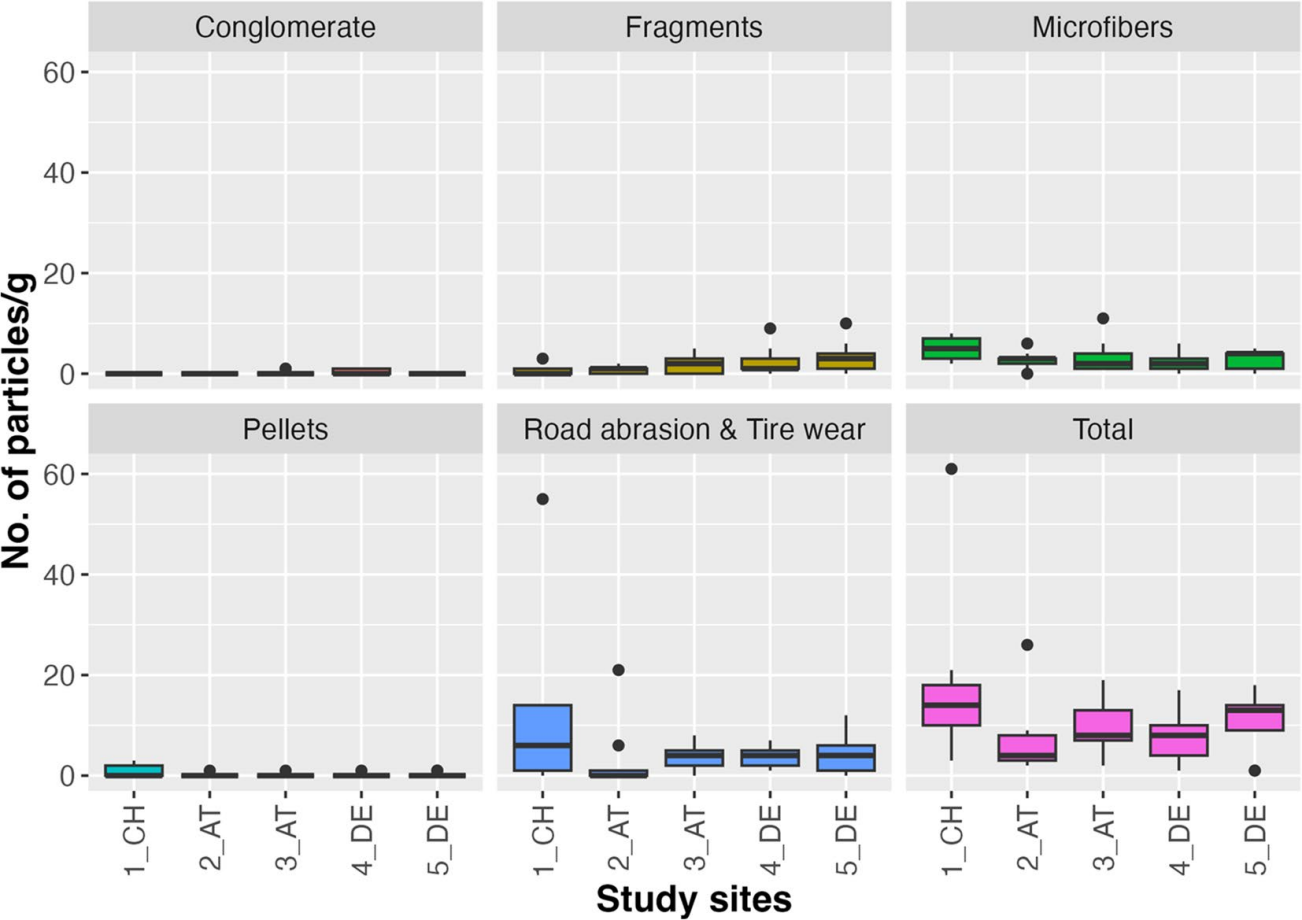
Boxplot of MP particle numbers per gram sample dry mass in the otter spraints (*n* = 45) at the different sampling sites along the River Inn, depicted for each particle category and for the total particle load (i.e., pooling of all particle types)

*Note*: Except for microfibers, particles that were suspected to be plastic according to the identification key by Lusher et al. (2020) were moved with metal tweezers to a metal container and stored for subsequent ATR FTIR analysis (Fig. 2). We ran ATR FTIR for 43 particles in question to identify the MP particles.

- *Step 1:* Filtering the suspension with a double stacked stainless-steel sieve system; 1 mm sieve stacked above a 20-μm sieve to separate bigger items (e.g., bones and scales) from smaller particles; rinsing the glass beaker with DI water for three times; the resulting permeate is discarded
- *Step 2:* Drying the sieves in an oven at 40–60 °C for at least 20 min before inspection under the microscope
- *Step 3:* Inspecting the items on the 1 mm sieve under the vis microscope (e.g., Olympus SZ51)
- *Step 4:* Manually discarding items remaining on the 1 mm sieve if they are clearly recognizable as fish scales, bones, insect remains or crustacean exoskeleton fragments (Public Lab, 2018); see also Figs. 2 and 3)
- *Step 5:* If an item was identified as a microfiber, application of the hot needle test (De Witte et al., 2014) to check if the fiber is synthetic or organic: A hot needle was placed in near vicinity of the fiber; in case that the fiber melted or curled, it could be categorized as MP, otherwise it could be assumed to be of natural origin. This test is not applicable to particles like fragments or pellets. For these particle categories, the MP identification key by Lusher et al. (2020) was applied (Fig. 2).

4) *Density separation*

Target: Separation of remaining, non-organic particles (e.g., sand and stones) from target particles

- *Step 1:* Placing the 20-μm sieve in a glass beaker, rinsing it thoroughly with sodium polytungstate (SPT, Roth, 8828) solution (density = 1.9 g/cm^3^). The high density of sodium polytungstate solution allows for separating geogenic particles from MP with comparatively high density, such as PET with a density of 1.38 g/cm^3^ or the lighter fraction of road abrasion and tire wear, which show densities between 1.5 and 2.2 g/cm.
- *Step 2:* Placing the glass beaker containing the sieve in an ultrasonic bath (e.g., TP690-A, Bioblock Scientific) for 10–20 s to detach particles from the sieve
- *Step 3:* Rinsing the sieve with polytungstate solution on both sides, removing the rinsed sieve from the beaker glass and transferring the sodium polytungstate solution from the glass beaker into a centrifuge tube; centrifuging at 4000 rpm for 25 min
- *Step 3:* Decanting the floating solution over a 20-μm sieve
- *Step 4:* Drying the sieve in an oven (40–60 °C) and inspecting the items on it under the vis microscope.

*Note*: Accounting for the higher probability of imperfect visual detection or identification of MP particles within this smaller particle fraction (Löder & Gerdts, 2015), additional methods like μ-FTIR analyses should be applied. In our case, 5 samples were analyzed via transmission FTIR.

### Measures to limit (cross-)contamination of samples in the laboratory

To avoid (cross-)contamination of samples during sample preparation and processing several measures were taken (see also Prata et al., 2019): We used (1) cotton lab-coats and cotton clothes, (2) glass petri dishes and glass beakers, and (3) aluminum foil for covering of petri dishes and beakers; (4) we regularly cleaned and wiped the work surfaces with water and a paper towel; (5) we cleaned all the equipment pre and post usage in an ultrasonic bath; (6) we avoided any plastic devices; in case of unavoidable plastic devices like pipette tips, airborne contamination of samples was minimized by opening the package only shortly before usage or washing of centrifuge tubes with deionized (DI) water prior to usage (Claessens et al., 2013). Addressing the hydrophobic surface characteristics of plastic devices, EtOH and water could be used sequentially to prepare plastic devices for further procedural steps. To monitor potential airborne contamination, we took blank samples (= lab blank samples). Thereby, an empty glass beaker is placed next to the ones containing otter feces and all procedural steps are taken, ultimately filtering it over a 20-μm sieve to check for particles.

### Chemical characterization of extracted MP particles

In the given study, we first focused on the detection, identification, and classification of MP particle types in spraint samples under the vis microscope (Bertling et al., 2022; Lusher et al., 2020). However, to address potential limitations in terms of imperfect detection and/or misclassifications, some additional Fourier transform infrared spectroscopic (FTIR) analyses were done (cf. Philipp et al., 2022). Depending on particle size, thickness, shape, and other features, different FTIR techniques might be applied for the chemical characterization of potential MP, like attenuated total reflectance (ATR) FTIR or micro-FTIR (μ-FTIR) (Prata et al., 2019). In the case of our spraint samples from the River Inn, 43 particles with a size > 500 μm were suspected as MP by applying the identification key of (Lusher et al., 2020) under the vis microscope. These particles were measured with ATR FTIR (Bruker ® Helios FTIR micro sampler (Tensor 27)). Up to three replicates of each sample were performed. The measurements were vector-normalized with the integrated software OPUS ® 7.2. selected spectral regions (i.e., 4000–2400 and 1800–400cm^-1^). The excluded spectral region (2400–1800 cm^-1^) contains no signals of any relevant functional group but only disturbing signals of the diamond crystal. One sample per sampling site (*n*_total_ = 5) was not analyzed via vis microscopy, but via transmission FTIR by the research institution OFI (Österreichisches Forschungsinstitut für Chemie und Technik) with a Perkin-Elmer spectrometer (Frontier) and a Perkin Elmer Spotlight 400 FTIR-Imaging System addressing particle fractions > 20 μm and < 700 μm. The samples were first filtered through a metal mesh (mesh size 100 μm) and then through a silicon filter (pore size 1 μm) and washed with ultrapure water. After drying (at RT), light microscopic images were taken at 7.5x magnification (whole filter) and 25x magnification (detailed images). The FTIR spectroscopic analyses were performed on the loaded Si filter in transmission imaging (1-μm pore size) and on the residues on the metal mesh (100-μm mesh size). The measurement parameters for the transmission imaging were: Resolution 8 cm^-1^, 25 μm pixel size, wavenumber range 650–4000 cm^-1^; light microscopic image of the Si filter (round, *d* = 11 mm) and the selected measuring area (~ 6.5 × 6.5 mm) with 49.2% > xx% > 26.5% analyzed filter surface; spectral image (60,000 individual spectra) after multicomponent analysis and correlation comparison with reference spectra. The software ‘Purency’ was used to detect potential microplastic spectra and compare them to references in OFI’s database.

### Recovery of MP

We assessed the MP recovery rate of the protocol based on spiking experiments using surrogate standards (see Philipp et al., 2022). Different types of surrogate MP particles were added to six test spraint samples with differing numbers (13 to 68) and combinations of MP particles per test sample (Table 1). To minimize potential confusion of spiked particles with previously existing particles in the spraints (i.e., false positive recovery), we used dyed particles and photo documentations of spiked particles. The spiked samples were subjected to the developed protocol (Fenton reaction, density separation) and inspected under the vis microscope for the added particles. By dividing the number of recovered particles by the number of spiked particles, we calculated the recovery of the individual MP types.

**Table 1:** Types, sizes, and distributors of surrogate standards spiked to the experimental samples, amount of surrogate standard particles added to individual spraint samples

Polymer type	Particle type	Distributor/origin	Sample number					
			*#1*	*#2*	*#3*	*#4*	*#5*	*#6*
Polyethylene terephthalate (PET) (3–5 mm)	Fragments (white)	PET-bottle (ground, sieved)	3	3	3	---	---	---
PET (25–60 μm)	Fragments (white)	PET-bottle (ground, sieved)	2	2	3	---	---	---
Polyethylene (PE) (53–63 μm)	Spheres (red)	Cospheric, CA, USA	---	---	---	55	53	63
Polypropylene PP (~ 5 mm)	Fragments	Goodfellow, Cooperation, PA, USA	---	---	---	---	---	1
PE (~ 5 mm)	Fragments	Goodfellow, Cooperation, PA, USA	---	---	---	---	---	1
Polypropylene (PP)	Fibers	Baumhueter Extrusion GmbH, DE	9	8	9	---	---	---
Artificial turf (~ 1 mm)			---	---	---	---	---	2
Tire wear (50–300 μm)	Tire wear (black)	Bundesanstalt für Gewässerkunde, DE	---	---	---	---	---	1
Total			**14**	**13**	**15**	**55**	**53**	**68**

## Results

### Recovery of spiked MP particles

Most surrogate particles that were spiked to the test samples were retrieved with a recovery rate of 100%, except for PET with particle sizes between 25 and 60 μm and PE-spheres (53–63 μm) (see Table 2). While the overall recovery rate of particles was 84.6 %, PET with particle sizes between 25 and 60 μm was only recovered in 28% of cases. The bigger the particles, the more likely they were to be found on the 1-mm sieve. The PE-spheres were usually found on the finer sieve (20 μm), but sometimes stuck to bones or scales and remained thus on the 1-mm sieve.

**Table 2:** Types and sizes of surrogate standard particles spiked to the experimental samples and referring recovery rates (in %) on the 1-mm and 20-μm sieves

Polymer type	Particle type	Recovery rate (%) on		Recovery rate (%) total
		1-mm sieve	20-μm sieve	
Polyethylene terephthalate (PET) (3–5 mm)	Fragments (white)	100		100
PET (25–60 μm)	Fragments (white)	14	14	28
Polyethylene (PE) (53–63 μm)	Spheres (red)	33	45	78
Polypropylene PP (~ 5 mm)	Fragments	100		100
PE (~ 5 mm)	Fragments	100		100
Polypropylene (PP)	Fibers (orange)	88	12	100
Artificial turf (~ 1 mm)		100		100
Tire wear (50–300 μm)	Tire wear (black)	100		100

### MP loads in field samples

MP were detected in all the 50 analyzed samples, and the particle types included pellets, fragments, microfibers, conglomerates, and road abrasion/tire wear (see Fig. S1 in Online Resource 1 and data in Online Resource 2).

During the visual classification under the vis microscope (applying the hot needle test (De Witte et al., 2014) and the identification keys of Lusher et al. (2020) and Public Lab (2018)), 512 MP particles were detected within the related sample set (*n* = 45; see data in Online Resource 2). Out of these, 43 particles with a size > 500 μm were suspected to be MP. Out of these 43 suspect particles, 7 particles were confirmed as MP (including polyethylene, polypropylene, polystyrol, and polyvinylchloride) by ATR FTIR measurements. The majority of the 43 suspect particles consisted of calcite (as typical for bones), other organic material or hair. Detected particles per sample were expressed as standard measure (i.e., number of particles per gram dry mass). Pooling these 45 samples of all study sites, up to 61 MP particles per gram spraint sample dry mass were observed with road abrasion/tire wear and microfibers being the most prominent particle types, both in terms of median and maximum particle numbers per gram spraint sample (Table 3).

**Table 3:** Number of detected particles per gram dry mass of spraint samples (*n* = 45; pooled across all study sites), indicated for each particle type and for the total MP load (i.e., pooling of all particle types). Median, minimum, and maximum values as well as mean and standard deviation are given per particle type and for the total MP load

	Number of particles				
Particle type	Median	Min	Max	Mean	Std Dev
Microfibers	3	0	11	3.3	2.4
Pellets	0	0	3	0.3	0.7
Fragments	1	0	10	1.8	2.3
Conglomerates	0	0	1	0.1	0.3
Road abrasion/tire wear	4	0	55	5.3	8.8
Total particle load*	**9**	**1**	**61**	**10.6**	**9.8**

*Sum of detected MP particles per gram dry mass when pooling all MP types

At study site level, we detected up to 11 microfibers per gram spraint sample dry mass and 55 road abrasion/tire wear particles (Fig. 3).

MP particles were detected within all five samples that were analyzed via transmission FTIR (Table 4).

**Table 4:** Results of measurements of the loaded Si filter in transmission imaging (1-μm pore size) *n* = 5

Sampling site	% of filter surface measured	No. and type of detected MP particles		
		Polyethylene PE	Polypropylene PP	Polystyrol PS
1-CH	38.7	3	---	---
2-AT	42.8	4*	1*	1
3-AT	49.2	13*	17*	---
4-DE	33.7	2	---	---
5-DE	26.5	2*	3*	---

*Partially mixed with polyamides PA

## Discussion

### Sample preparation protocol—MP extraction and chemical characterization

Following the review of Eerkes-Medrano et al. (2015) several criteria should be considered when targeting at the development of effective methods for the detection of MP for practical monitoring contexts: Methods should be simple and quick to allow for enough replications to capture the variability between different samples. The costs should be low enough that the method can actually be used in a practical context. The methods should be both precise and accurate and contamination should be limited as much as possible. However, the optimal selection and combination of methods and single procedural steps have to balance pros and cons of distinct methodical approaches and it is highly context-dependent. In the following, we discuss selected steps of our protocol in terms of Eerkes-Medrano et al.’s (2015) criteria.

We used oxidative reaction (Fenton reaction) to digest the non-target organic material in the otter spraints allowing for the subsequent visual identification of MP particles under the vis microscope. Fenton reaction is one widely used digestion method for different matrices (Al-Azzawi et al., 2020; Masura et al., 2015; Sarkar et al., 2023), outperforming other digestion procedures for substrates like sludge and soil (Hurley et al., 2018) that are comparable with otter spraints. Philipp et al. (2022) showed that polylactide (PA) fibers might change in physical properties (i.e., milky appearance of the surface and brittleness) when being subjected to Fenton reaction, while other tested polymers (PET, PP, PE, PMMA, and PVC) remained unaffected by this method. This is in line with earlier studies on impacts of Fenton reaction on MP (e.g., Hurley et al., 2018; Li et al., 2018; Sujathan et al., 2017). Alternatively, enzymatic digestion is frequently used in microplastic research (Karlsson et al., 2017). However, this method requires much more time and has been shown to damage certain MP types (Al-Azzawi et al., 2020) while not reliably removing all organic matter (Lusher et al., 2020). A different approach has been used by Winkler et al. (2020) who used visual analysis and only applied density separation to detect microplastics in kingfisher (*Alcedo atthis*) pellets, which mainly consist of bones and hardly contain other organic material. When Smiroldo et al. (2019) found the first microplastic items in otter spraints, the samples were soaked in hydrogen peroxide for 24 h prior to analysis. This study however did not aim at finding microplastics but at analyzing the otters’ diet. A further crucial step in MP extraction is density separation (i.e., the separation of sand, stones, etc. from target MP particles). This can be done by using density solutions (sodium chloride, sodium bromide, zinc chloride, or sodium polytungstate), or applying elutriation-based or ultrasonic methods (Claessens et al., 2013; Klöckner et al., 2019; Sarkar et al., 2023; Zhu, 2015). We applied density separation for the smaller particle fraction (on the 20-μm sieve) using sodium polytungstate (SPT, Roth, 8828) solution with a relatively high density of 1.9 g/cm^3^. High density is required for an effective density separation of particular polymer types like road abrasion and tire wear, which might show densities between 1.5 and 2.2 g/cm^3^ (road abrasion; Kayhanian et al., 2012) and approximately 1.2 g/cm^3^ (tire wear; Degaffe & Turner, 2011). Density separation with sodium polytungstate solution proved to separate tire wear and road abrasion particles both in our spiking experiment and in the field samples. As tested by Klöckner et al. (2019) more than 90% of tire and road wear particles can be separated from a sample matrix by using a sodium polytungstate solution with a density of 1.9 g/cm^3^. Although the density of polytungstate solution can be adjusted to higher values (up to 3.1 g/cm^3^), it is comparatively expensive and based on the use of a heavy metal. Both facts are in contradiction with Eerkes-Medrano et al.’s (2015) criteria for the development and choice of MP research techniques. An alternative could be NaI solution with a density of 1.8 g/cm^3^ (Hurley et al., 2018), but again carrying the risk of losing particles with higher densities.

Applying the Fenton reaction and a density separation with sodium polytungstate solution to test spraints spiked with MP particles in our study resulted in a total recovery rate of 85% when pooling all particle types and 100% recovery rate for most tested particle types. However, these rates were not only driven by the method for sample preparation but also by the (visual) detection method. In particular, recovery rates of spiked particles were unsatisfactory for the smaller PET fraction. This clearly shows the necessity of further analytical steps in addition to visual analyses, as was also done to a limited extent in the present study. Correctly identifying particles as MP when exclusively relying on visual analysis remains challenging as the shape and size of particles vary widely. The success of the visual technique is therefore highly dependent on the researchers’ experience and equipment (cf. Meixner et al., 2020). Error rates from 20% (Lenz et al., 2015) to 70% (Hidalgo-Ruz et al., 2012) have been observed and are assumed to increase with decreasing particle size (Löder & Gerdts, 2015). The manual from Lusher et al. (2020) for the visual classification of MP together with the hot needle test (De Witte et al., 2014) and the identification guide by Public Lab (2018) provide a good basis for reducing misidentifications. Visual classifications also allow for a determination of different particle shapes and of combinations of particle types (particularly conglomerates), which might not be provided by other procedures. However, accounting for potential detection and classification failures during visual analyses under the vis microscope (Eriksson et al., 2013), the application of other additional analytical methods like FTIR or micro-Raman spectroscopy is frequently recommended (Hidalgo-Ruz et al., 2012; Löder & Gerdts, 2015). In our study, we laid the main focus on visual analyses of spraint samples accounting for Eerkes-Medrano et al.’s (2015) criteria of simplicity and affordability for method selection. In our study, both ATR FTIR and transmission FTIR analyze proved to be meaningful additional procedural steps counterbalancing potential weaknesses of solely visual analyses, albeit being expensive. As μ-FTIR analyses are usually limited to sections of a filter (Löder & Gerdts, 2015), they also hold some risk of missing MP particles at other sections and omission rates will depend on the number of screened filter sections. Summing up, a combination of methods, e.g. visual inspection under the vis microscope together with FTIR analyzes (cf. Alvarez-Aquino et al., 2004; Löder & Gerdts, 2015; Lusher et al., 2020; Philipp et al., 2022) or other analytical methods (e.g. pyrolysis-gas chromatography–mass spectrometry (Py-GC/MS)) is recommended (e.g., Goßmann et al., 2021; Kamp et al., 2023; Meixner et al., 2020; Prata et al., 2019) if higher costs are acceptable in favor of accuracy. As recommended by the MSFD technical subgroup on Marine Litter (Hanke et al., 2013), 10% of MP with a size range between 100 μm and 5 mm should be analyzed using FTIR techniques, and all suspected particles with a size range between 20 and 100 μm.

### MP loads in otter spraints along the River Inn

In this study, we provide further evidence of the occurrence of MP particles in Eurasian otter spraints with a prevalence of 100% within our samples. Considering the visually analyzed samples, we found an average of 10.6 ± 9.8 particles per g dry weight. This exceeds the number of MP particles reported by O’Connor, Lally, Mahon, et al. (2022) in otter spraints from Ireland, who reported 3.8 ± 0.6 particles per 1 g dry weight. However, O’Connor, Lally, Mahon, et al., 2022 omitted road abrasion particles, which made up almost 50% of all particles found in otter spraints from the River Inn in this study. As the most abundant MP category, road abrasion particles were present at all five sampling sites and in 80% of the samples. As already stated by Bertling et al. (2022), road abrasion and tire wear represent the most important source of MP emission into the environment. The second most abundant MP category in our data set were microfibers. These particles are entering the environment through the wear and washing of synthetic clothes (Gaylarde et al., 2021) and have also been reported as highly abundant in freshwater systems (Dris et al., 2018) and also in otter feces (O’Connor, Lally, Mahon, et al. (2022)). At two sampling sites, we found “conglomerates,” a MP category which is not often addressed. In the case of otters, conglomerates consisted of hard prey remains (e.g., fish bones and scales) that were held together by several microfibers. Whereas single MP particles might get excreted more easily, a larger indigestible construct in form of conglomerates (here with a size of up to 500 μm) might pose a risk of being stuck in the digestive tract potentially causing health issues.

## Conclusion

Applying our protocol for MP extraction and analysis, high recovery rates for most tested particle types could be achieved within a spiking experiment with otter spraint test samples. However, for smaller PET fragments, our approach was not completely satisfying. Hence, further analytical steps in addition to visual analyses, and a combination of methodological approaches, e.g., visual inspection under the vis microscope together with FTIR analyzes, is strongly recommended.

Within our small field study, we could show that the occurrence of MP in otter spraints is widespread. However, its origin and potential drivers remain poorly investigated. Here, we can only assume potential drivers of MP loads in otter spraints (e.g., land use, road and traffic density, human population density, wastewater treatment plants), but further investigations are needed to identify the most influential factors, considering also the complexity of food webs. Furthermore, otters can be used as sentinel species for MP pollution in aquatic ecosystems, and future studies should focus on the consequences of the ingestion and digestion of MP by otters, especially in the light of species conservation.

### Supplementary information

ESM 1 (DOCX 19760 kb)

ESM 2 (XLSX 11 kb)

## Data Availability

Not applicable.
