# Supplementary material for: Microplastic loads in Eurasian otter (Lutra lutra) feces—targeting a standardized protocol and first results from an alpine stream, the River Inn
Source: Environ Monit Assess. 2024 Jul 6;196(8):707. doi: 10.1007/s10661-024-12791-z (PMC11227469; doi:10.1007/s10661-024-12791-z)
Supplement: Supplementary file 1 — (DOCX 19760 kb) [file 10661_2024_12791_MOESM1_ESM.docx]

**Online Resource 1**

Article Title: Microplastic loads in Eurasian otter (*Lutra lutra*) feces – targeting a standardized protocol and first results from an alpine stream, The River Inn

Journal Name: Environmental Monitoring and Assessment

Authors: Nopp-Mayr U, Layendecker S, Sittenthaler M, Philipp M, Kägi R, Weinberger I

Corresponding Author: Sittenthaler M, [marcia.sittenthaler@boku.ac.at](mailto:marcia.sittenthaler@boku.ac.at)

**Table S1** Characteristics of the River Inn at the five otter feces sampling sites in Switzerland (CH), Austria (AT) and Germany (DE) (longitudinal zonation according to Illies (1961))

| Sampling site | Nearby city | Altitude  (m a.s.l.) | River width (m) | Mean discharge MQ (m^3^/s) | Longitudinal zonation of running waters |
| --- | --- | --- | --- | --- | --- |
| 1-CH | Samedan Bever | 1716 | 10 | 5 | Epi-/meta-rhithral |
| 2-AT | Prutz | 868 | 64 | 21 | Hypo-rhithral |
| 3-AT | Innsbruck | 569 | 66 | 163 | Hypo-rhithral |
| 4-DE | Rohrdorf | 454 | 102 | 390 | Hypo-rhithral |
| 5-DE | Simbach am Inn | 336 | 250 | 740 | Epi-potamal |

| **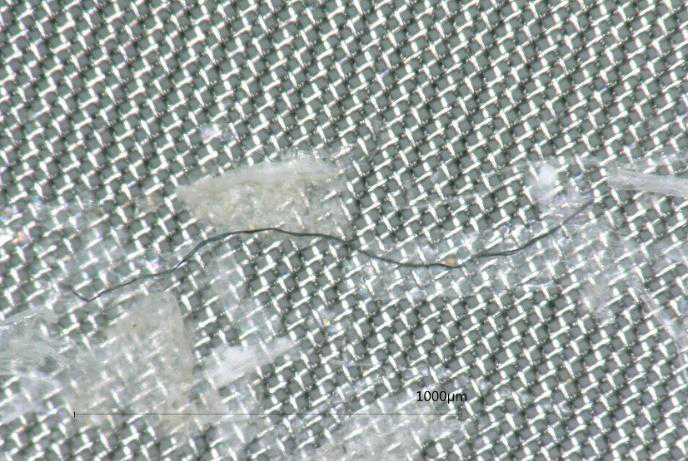**  **1000 μm** | (a) | **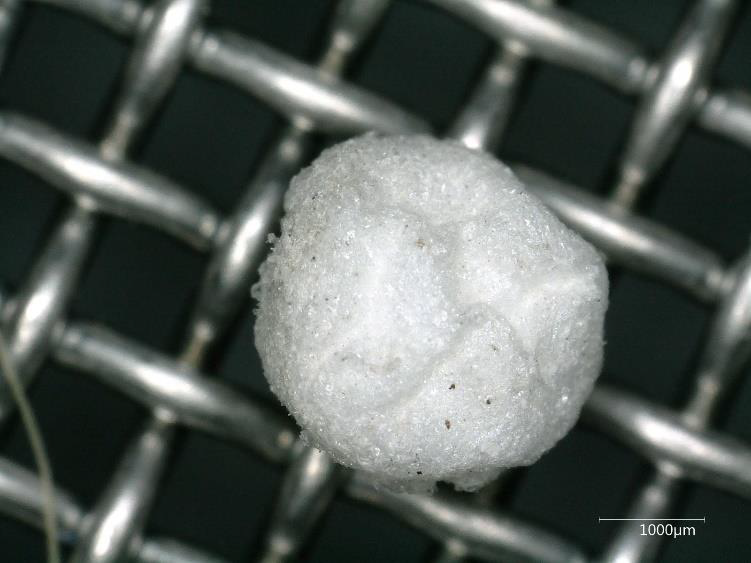**  **1000 μm** | (b) |
| --- | --- | --- | --- |
| **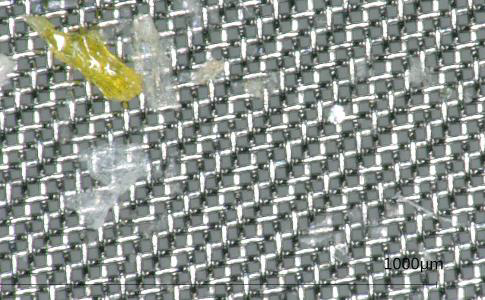**  **1000 μm** | (c) | **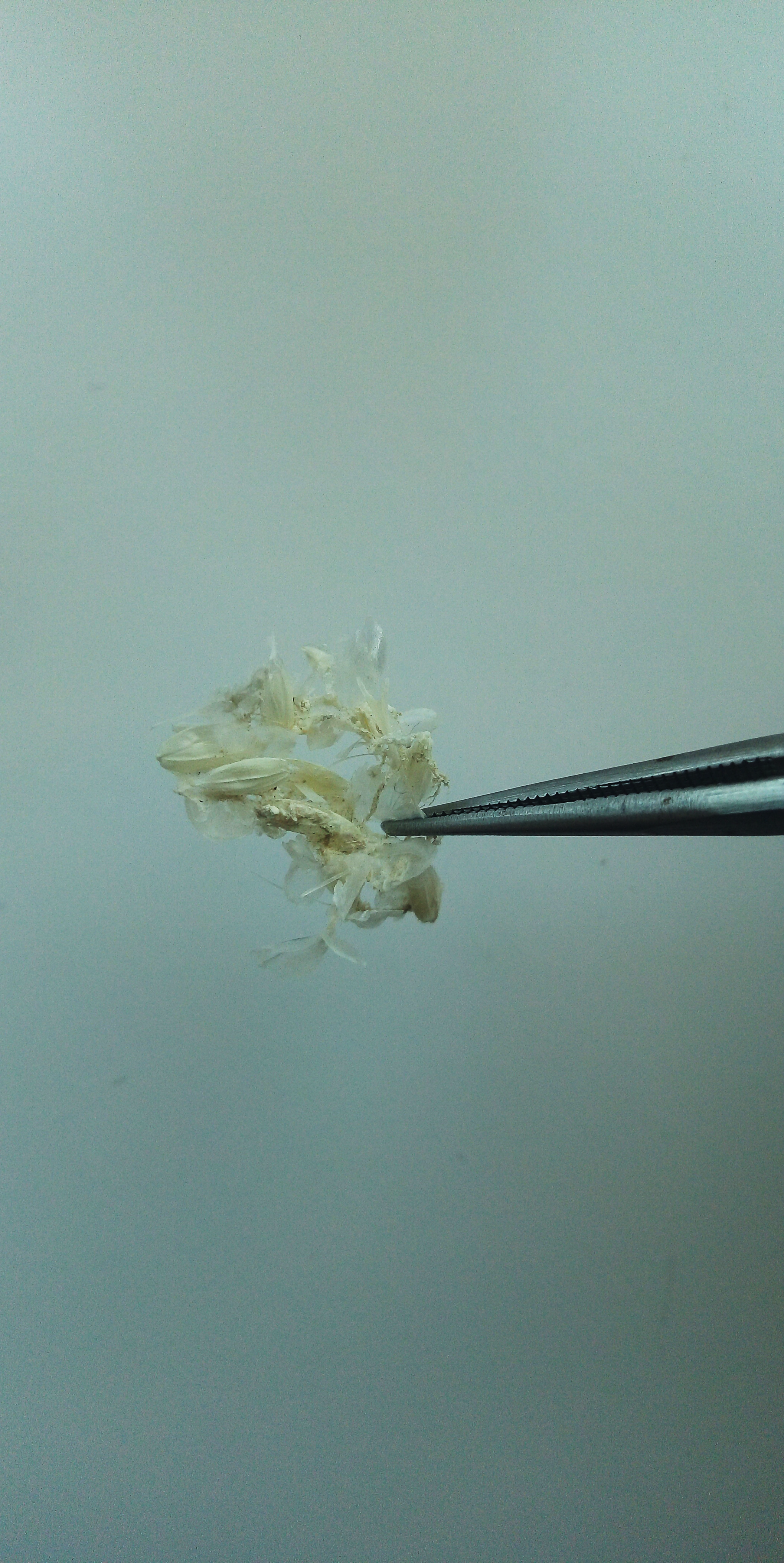**  **5000 μm** | (d) |
| **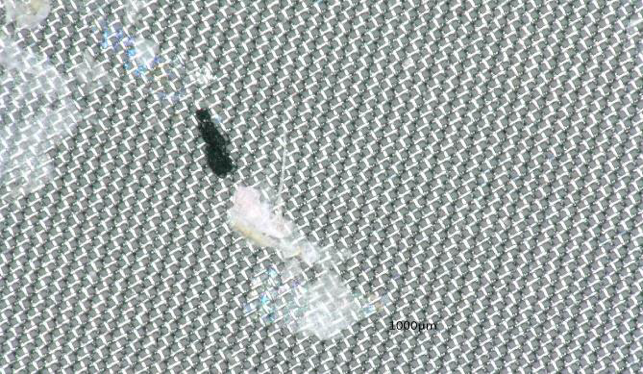**  **1000 μm** | (e) | **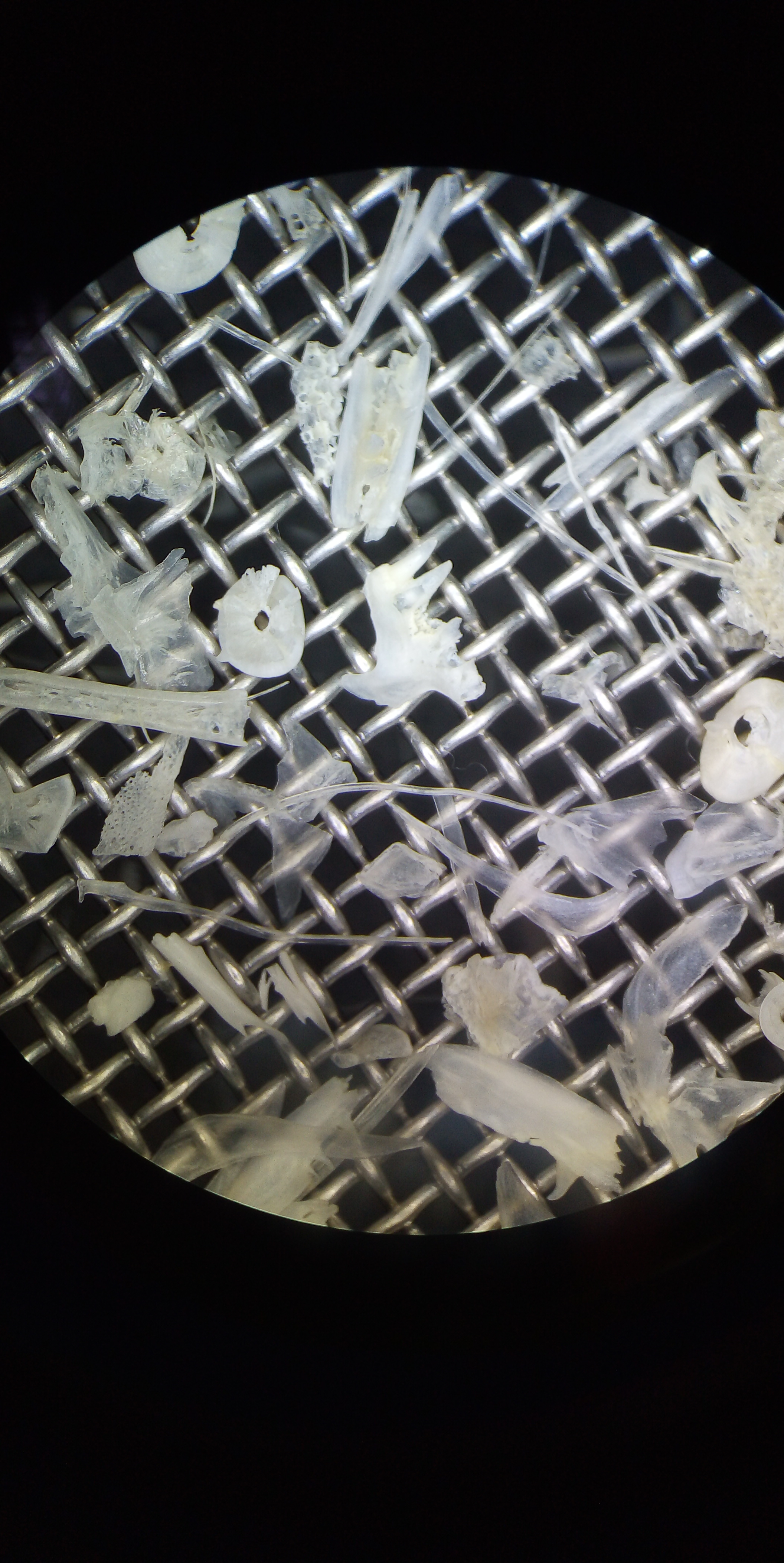**  **1000 μm** | (f) |

**Fig. S1** Pictures of microplastic particles in the otter spraints from the sampling sites along the River Inn (a: microfiber, b: pellet, c: fragment, d: conglomerate, e: road abrasion); (f) sample section showing typical hard prey remains in otter spraints without the presence of microplastic

**References**

Illies, J. (1961). Versuch einer biozönotischen Gliederung der Fließgewässer, 46 (2), 205–213.
